# Optimized Extraction Protocols for Bioactive Antioxidants from Commercial Seaweeds in Portugal: A Comparative Study of Techniques

**DOI:** 10.3390/foods14030453

**Published:** 2025-01-30

**Authors:** Francisca Santos, Cristina Soares, Stephanie L. Morais, Cátia Neves, Clara Grosso, Maria João Ramalhosa, Mónica Vieira, Cristina Delerue-Matos, Valentina F. Domingues

**Affiliations:** 1 REQUIMTE/LAQV, Instituto Superior de Engenharia do Porto, Instituto Politécnico do Porto, Rua Dr. António Bernardino de Almeida 431, 4249-015 Porto, Portugal; ffpls@isep.ipp.pt (F.S.); stlom@isep.ipp.pt (S.L.M.); 10200688@ess.ipp.pt (C.N.); claragrosso@graq.isep.ipp.pt (C.G.); mjr@isep.ipp.pt (M.J.R.); cmm@isep.ipp.pt (C.D.-M.); 2 Chemical and Biomolecular Sciences, School of Health (ESS), Polytechnic of Porto, 4200-465 Porto, Portugal; 3 RISE-Health, Center for Translational Health and Medical Biotechnology Research (TBIO), CQB, ESS, Polytechnic of Porto, R. Dr. António Bernardino de Almeida, 400, 4200-072 Porto, Portugal; mav@ess.ipp.pt

**Keywords:** antioxidant capacity, macroalgae, solid–liquid extraction, subcritical water extraction, ultrasound-assisted extraction

## Abstract

This study aimed to optimize the extraction conditions for a valuable source of antioxidants: seaweed. Therefore, ten seaweed samples were subjected to a solid–liquid extraction (SLE), where the extraction conditions (biomass (g): solvent (mL) ratio, temperature, and time) were optimized using response surface methodology (RSM). The seaweeds were also subjected to subcritical water extraction (SWE) (140 and 190 °C) and ultrasound-assisted extraction (UAE) (10 and 20 min). The antioxidant capacity of the extracts was determined through the ferric-reducing antioxidant power and the 2,2′-azino-bis(3-ethylbenzothiazoline-6-sulfonic acid). The total phenolic content revealed the significance of temperature and biomass; solvent ratio parameters in the extraction process with higher conditions generally promoting the release of phenolic compounds. Furthermore, applying RSM allowed for the identification of optimal conditions and the establishment of predictive models that can be valuable in industrial-scale extraction processes. The antioxidant potency composite index (APCI) shows that SWE at 190 °C stands out, with *E. bicyclis* reaching an APCI score of 46.27%. The AGREEprep evaluation showed that UAE is the most sustainable method, achieving the highest score (0.69). The results of this study contribute to the development of efficient and standardized extraction protocols for each seaweed species, allowing for the maximum yield of antioxidants.

## 1. Introduction

Macroalgae, or seaweeds, are a diverse group of photosynthetic organisms that play an essential role in aquatic ecosystems and are recognized as a valuable source of fibers, minerals, and moderate levels of proteins and lipids (omega-3 fatty acids), making them nutritionally important for human consumption [1,2,3,4,5]. Depending on their pigmentation, these organisms are classified into three main groups: green (*Chlorophyta*), brown (*Phaeophyta*), and red (*Rhodophyta*) algae.

Brown algae vary in color from olive green to dark brown and can grow up to 100 m long. Around 1500 known species are found in polar locations, such as North America, Europe, the Gulf of Mexico, and the mid-Atlantic. In addition, they have the most complex stem structure, are leathery, and can withstand exposure to air. They prefer shallow, cold waters and thrive on rocky shores. In terms of their constitution, they contain chlorophyll A and C and fucoxanthin and have algin and pectin cell walls [6,7,8].

Green algae are multicellular organisms with a simple stem structure and the appearance of filamentous spongy fingers or paper-thin leaves. These algae consist of between 700 and 7000 species and are usually found in bays, estuaries, and tidal pools. Green algae contain chlorophylls A and B and carotenoids, and store excess energy in starch, with cellulose in their cell walls [6,7,8].

On the other hand, red algae, which grow as filaments or layers of cells, include parasitic forms of other algae. There are around 4000 species, making them the most abundant and widespread group of algae. These algae prosper in various environments, from deep, cold waters to warm, shallow seas. They contain chlorophyll A and phycobilins and often have calcium carbonate in their cell walls, such as the algae *Gracilaria verrucosa*, *Palmaria palmata,* and *Asparagopsis armata* and are, thus, called corallines [6,7,8].

Seaweed is a rich source of health-promoting compounds, particularly antioxidants, which help neutralize free radicals and reduce oxidative stress in the body [1,2,5,9]. These properties make seaweed and seaweed-derived products highly beneficial for human health. Additionally, research has demonstrated the effectiveness of seaweed antioxidants in preventing lipid peroxidation, highlighting their potential application as natural antioxidants in the food, cosmetic, and pharmaceutical industries [5,10]. This has attracted significant interest from researchers and industries seeking to exploit their health benefits.

Both conventional and advanced extraction methods have been employed to isolate these antioxidants efficiently. Conventional methods, such as solid–liquid extraction (SLE), are widely used due to their simplicity, while advanced techniques, including ultrasound-assisted extraction (UAE) and subcritical water extraction (SWE), offer improved efficiency and yield [11,12]. Beyond these, several alternative methods have been explored in the literature to enhance the extraction of total phenolic compounds (TPC) and antioxidants (measured by FRAP) from seaweeds. One promising approach is supercritical fluid extraction (SFE), which uses supercritical fluids, such as carbon dioxide, to extract target compounds with reduced solvent use, shorter extraction times, and higher selectivity. Similarly, pressurized liquid extraction (PLE) showed notable results in a study by Tierney et al. [13]. Tierney et al. [14] reported higher yields of phenolic content and antioxidant activity with PLE compared to conventional methods [14].

Nonetheless, extracting these bioactive compounds from seaweed is challenging due to the variety of compounds and seaweed species, each requiring specific extraction conditions to optimize yield and quality [15]. The extraction of antioxidants from seaweed usually involves pre-treatment, extraction, and separation steps, with conditions like solvent choice, temperature, and time having a significant impact on the result [16]. So, optimizing the extraction conditions is crucial to maximizing the benefits of seaweed antioxidants.

Response surface methodology (RSM) is a widely used statistical tool for optimizing extraction conditions and enhancing yields while minimizing resource use. By modeling variables such as temperature, time, and solvent concentration simultaneously, RSM provides valuable insights into the interactions of the factors that are critical for efficient extraction [17,18]. RSM has been widely used to optimize the extraction conditions for bioactive compounds from seaweed. For example, RSM was applied to extract reducing sugars and polyphenolic compounds from *Rugulopteryx okamurae* (E.Y. Dawson) I.K. Hwang, W.J. Lee, and H.S. Kim, 2009, optimized key parameters, such as drying temperature, milling time, and solvent type [19]. Another study used RSM to enhance the yield of antioxidants, phenolic compounds, and fucoxanthin from *Padina australis* Hauck 1887 under optimized solvent concentration, temperature, and time [20]. Similarly, supercritical fluid extraction combined with RSM was employed to extract fucoxanthin from the waste stems of *Undaria pinnatifida* (Harvey) Suringar, 1873, achieving high yields while valorizing the byproducts [21]. These examples demonstrate RSM’s effectiveness in optimizing seaweed bioactive extractions.

Despite some limitations, RSM has proven effective in refining extraction protocols, reducing experimental trials, and ensuring reliable outcomes [22,23]. The selection of an extraction method for RSM optimization must consider the ability to control key variables and the energy demands associated with each technique. Advanced methods like SWE and UAE, while effective at extracting bioactive compounds, are highly energy-intensive. SWE requires elevated temperatures and pressures to maintain subcritical conditions, while UAE relies on high-power ultrasonic waves, which lead to substantial energy consumption. Given that RSM requires numerous experimental runs to systematically evaluate variable effects, using energy-intensive methods like SWE or UAE would result in impractical energy demands and higher operational costs.

In contrast, SLE operates under milder conditions, requires less energy, and uses simpler equipment, making it more suitable for extensive optimization studies [24,25]. SLE also allows for the simultaneous processing of multiple samples, unlike SWE and UAE, which are constrained to single-sample processing due to their operational setups. This efficiency further supports the practicality of SLE for RSM optimization studies. Previous research has demonstrated the successful use of SLE combined with RSM for optimizing the extraction of phenolic compounds and antioxidants from various natural matrices [24,25].

**Figure 1:**
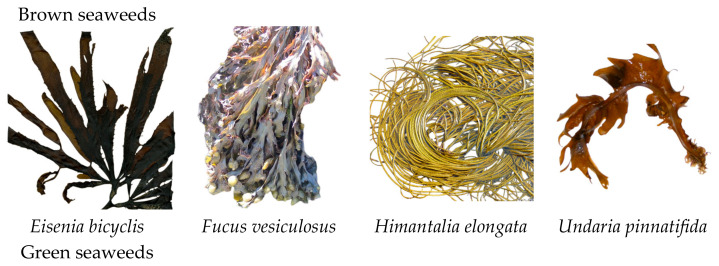

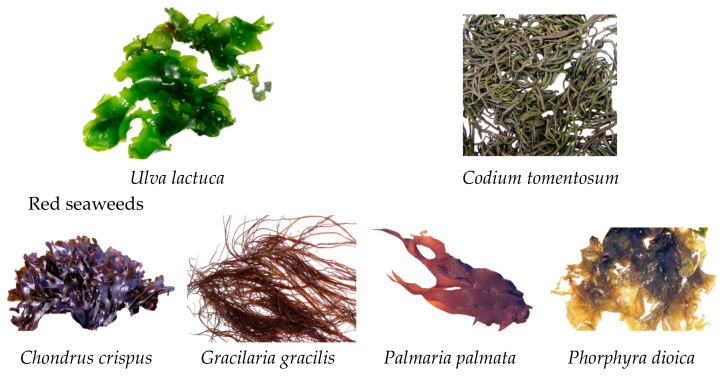
Selected brown, green, and red seaweeds.

In this study, SLE was initially performed on ten commercially available seaweed species in the Portuguese market. These included four brown seaweeds (*Eisenia bicyclis* ((Kjellman) Setchell, 1876, *Fucus vesiculosus* Linnaeus, 1753, *Himanthalia elongata* (Linnaeus) S.F. Gray, 1821 and *U. pinnatifida*), two green seaweeds (*Ulva Lactuca* Linnaeus, 1753 and *Codium tomentosum* Stackhouse, 1797), and four red seaweeds (*Chondrus crispus* Stackhouse, 1797, *Gracilaria gracilis* (Stackhouse) Steentoft, Irvine et Farnham, 1792, *Palmaria palmata* (Lineu) F. Weber and D. Mohr 1891 and *Phorphyra dioica* J. Brodie and L.M. Irvine, 1997) (Figure 1). All seaweeds have unique characteristics and potential antioxidant properties. These seaweeds were chosen for their unique characteristics and potential antioxidant properties. The optimization process focused on SLE and three key parameters, namely temperature, biomass-to-solvent ratio (g/mL), and time. Based on these parameters, RSM was applied to determine the optimal extraction conditions for SLE. The optimized biomass-to-solvent ratio was subsequently applied to two advanced extraction techniques: SWE and UAE.

This approach allowed for the evaluation of SWE and UAE under pre-optimized conditions, thus reducing the number of experimental setups while ensuring an efficient recovery of phenolic compounds and antioxidants. The objective was to identify the optimal operational parameters for each extraction method, facilitating comparisons and optimization. Statistical designs, such as Box–Behnken and central composite designs, were employed to systematically evaluate the extraction efficiency in SLE before extending the optimized conditions to SWE and UAE. Furthermore, the greenness of each technique was assessed to develop protocols that are both environmentally sustainable and suitable for industrial applications.

## 2. Materials and Methods

### 2.1. Materials

Sodium carbonate, Folin–Ciocalteu reagent, 2,4,6-Tri-(2-pyridyl)-s-triazin (TPTZ) reagent, ascorbic acid, iron (III) chloride, hydrochloric acid, glacial acetic acid, potassium persulfate, and 2,2-azinobis (3-ethylbenzothiazoline-6-sulfonic acid) diammonium salt (ABTS) were purchased from Merck (Darmstadt, Germany); gallic acid and Trolox were from Honeywell Fluka (Buchs, Switzerland). Sodium acetate was from Panreac (Barcelona, Spain). All chemicals were used without further purification.

### 2.2. Identification and Sampling

In this study, ten seaweeds were selected: four brown seaweeds (*E. bicyclis*, *F. vesiculosus*, *H. elongata*, and *U. pinnatifida*), two green seaweeds (*U. lactuca* and *C. tomentosum*), and four red seaweeds (*C. crispus*, *G. gracilis*, *P. palmata*, and *P. dioica*). All seaweed species were acquired as commercial dried and ready-to-eat ingredients from different brands available in natural products stores in Porto, Portugal.

Before the extraction, all samples were rehydrated in a 35 g/L NaCl solution for 5 min, then rinsed with deionized water to remove the excess salt. The 10 seaweeds were then dehydrated at 41 °C (Excalibur 9 Tray Dehydrator, Model 4926 T, Barcelona, Spain) for 24 h and minced in a Moulinex grinder (Paris, France).

The ground samples were stored in a dark and dry place until further use.

### 2.3. Antioxidants and Bioactive Compounds Extraction

At this stage, all the algae were subjected to three different extraction techniques: SLE, SWE, and UAE.

SLE was selected for the RSM optimization study due to its ability to process multiple samples simultaneously, its precise control of variables such as temperature, time, and biomass-to-solvent ratio, and its simplicity and cost-effectiveness. In contrast, SWE and UAE are limited to processing one sample at a time due to equipment constraints, and their additional variables, such as pressure and ultrasonic amplitude, could complicate modeling. These factors make SLE ideal for statistical modeling and predictive equation development.

**Figure 2:**
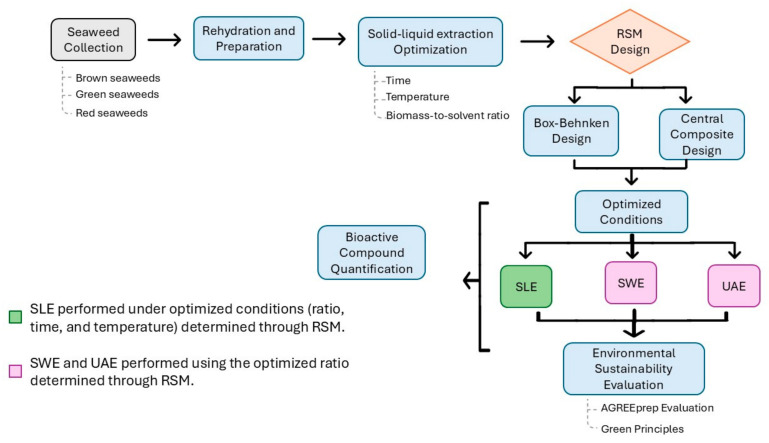
Flowchart summarizing the optimization and evaluation of seaweed extraction techniques, integrating RSM design, bioactive compound quantification, and sustainability assessment. SLE—solid–liquid Extraction; SWE—subcritical water extraction; UAE—ultrasound-assisted extraction.

The optimized extraction parameters obtained from the SLE RSM study were subsequently applied to SWE and UAE to evaluate their efficiency. After extraction, all algae extracts were lyophilized and stored in a dark, dry place for further analysis. The experimental procedure applied to the seaweed is illustrated in Figure 2.

#### 2.3.1. Experimental Design

SLE was employed for optimization using response surface methodology (RSM). Two statistical models were applied: the Box–Behnken design (BBD) and the central composite design (CCD). BBD is suitable for estimating quadratic model parameters, enabling a comprehensive analysis of variable relationships. CCD, comprising factorial, axial, and central runs, was an alternative RSM optimization tool.

The experimental data were analyzed using Design-Expert 11.0.0 software to determine the optimal extraction conditions. The fixed parameters included (1) solvent type: deionized water; (2) stirring speed: 200 rpm; and (3) solvent volume: 40 mL.

The key variables—temperature, biomass-to-solvent ratio, and time—were varied within specific ranges selected based on the literature [26,27].

The CCD model (S1) included 17 parameter combinations to ensure statistical validity, while the BBD model (S2) used 15 combinations.

The optimized conditions derived from the SLE study were later compared to those of SWE and UAE.

#### 2.3.2. Solid–Liquid Extraction (SLE)

The SLE was conducted using several factors that were carefully controlled during the extraction process, including temperature, seaweed mass-to-solvent ratio, and extraction time, which were selected based on the data available in the literature [26,27].

Regarding the ratio, preliminary tests were performed to evaluate mass-to-solvent ratios ranging from 1:5 to 1:100. A one-way ANOVA and Tukey’s post hoc test were conducted to identify significant differences in TPC and FRAP values across the ratios. Based on the statistical results and observed trends, 1:25, 1:50, and 1:75 (grams of seaweed to milliliters of solvent) ratios were selected for optimization, showing consistently high responses while minimizing solvent use.

Three different extraction times were employed: 1 h, 3 h, and 5 h. Three temperature levels (25 °C, 50 °C, and 75 °C) were considered key factors in the extraction procedure. Three replicates were performed for each model, with 17 trials for the CCD and 15 for the BBD, per the response surface methodology (RSM) models.

Each tested seaweed was placed in an Erlenmeyer flask covered with aluminum foil to prevent solvent evaporation at higher temperatures. The Erlenmeyer flasks were placed on a hot plate magnetic stirrer to maintain the desired temperature and with constant homogenization at 200 rpm (VWR, Model VMS—C7, Carnaxide, Portugal).

The seaweed samples were in direct and constant contact with the solvent, releasing the antioxidants from the matrix into the liquid phase, facilitating the efficient extraction of the antioxidants, and allowing subsequent analysis and characterization of these bioactive compounds. The samples were filtered through gauze, and the mixture obtained was lyophilized and stored until further use.

#### 2.3.3. Subcritical Water Extraction (SWE)

The SWE used 300 mL of water in a subcritical extraction system (Parr Instrument Company, Model 4848, Moline, IL, USA). In this extraction, the seaweeds were subjected to two extractions at different temperatures (140 °C, 20 bar, and 190 °C, 30 bar), in which two replicates were carried out. Both extractions were performed in 30 min. The selection of these parameters was based on the data available in the literature to ensure the application of conditions known to improve the extraction efficiency of bioactive compounds [28,29,30].

The ratio of seaweed mass to solvent used was 1:75 for all the seaweeds, except for *U. lactuca* and *U. pinnatifida*, where the ratio was 1:100.

The samples were filtered through gauze, and the resulting mixture was lyophilized and stored for future use.

#### 2.3.4. Ultrasound-Assisted Extraction (UAE)

UAE was carried out using 75 mL of water. The ultrasonic processor (Sonics & Materials, Model CV334, Newtown, CT, USA) was utilized with an amplitude of 40% at 20 °C, and two extractions were carried out at 10 min and 20 min. Each extraction condition was conducted in duplicate. These parameters were chosen based on the literature, ensuring the conditions used demonstrated optimization of the extraction efficiency of bioactive compounds [27,30]. Each seaweed was placed in an ice bath to prevent the mixture’s temperature alteration.

As with the SLE and SWE, the solvent in this extraction was in direct contact with the seaweed samples, allowing for a more effective extraction. For both extraction conditions, the samples were then filtered through gauze, and the liquid obtained was stored in plastic containers and frozen at −80 °C for lyophilization.

### 2.4. Analysis of Phenolic and Antioxidant Content

#### 2.4.1. Total Phenolic Content

Total phenolic compounds (TPC) were determined as described by Barroso et al. [31]. First, six gallic acid (GA) standards were prepared to construct the calibration curve, with concentrations ranging from 10.00 to 200.00 µg of GA/mL. Then, in a microplate, 25 μL of each standard and sample, in triplicate, were added to 75 μL of water in each well, totaling 100 μL. Afterward, 25 μL of Folin–Ciocalteu reagent were added, and the mixture was incubated for 6 min in the dark. After incubation, 100 μL of sodium carbonate were added, and the reaction was kept in the dark for 1 h. Finally, the samples were analyzed at 765 nm using a Synergy HT microplate reader (Biotek Instruments, Winooski, VT, USA). The total phenolic content was then expressed in mg of gallic acid equivalents per g of seaweed extract dry weight (mg GAE/g d.w.).

#### 2.4.2. Ferric-Reducing Antioxidant Power (FRAP) Analysis

The FRAP test was carried out as described by Barroso et al. [31]. In general, the FRAP reagent was prepared daily with previously prepared solutions of acetic acid buffer (300 mM), FeCl3 (20 mM in dist. water), and TPTZ (10 mM in HCl (40 mM)) in a ratio of 10:1:1 (*v*/*v*/*v*). In addition, ascorbic acid (AA) was used as a standard solution for this measurement, and several dilutions were prepared to construct a calibration curve, with concentrations ranging from 5.00 to 100.00 µg of AA/mL. Next, 20 μL of the standard and sample were placed in a microplate, followed by 180 μL of FRAP reagent, except for the blank, which only had 200 μL of deionized water. It was then shaken for 10 min and read at 593 nm at 37 °C using a BioTek Synergy HT plate reader. The FRAP value was calculated using Equation (1) and expressed in mg of ascorbic acid per g of seaweed extract dry weight (mg AA/g d.w.).

(1) $${FRAP}_{value}=\frac{A_{1}-A_{0}}{A_{c}}$$

where

${Abs}_{1}$—absorbance of the sample at 593 nm.

$A_{0}$—absorbance of the intercept at 593 nm.

$A_{c}$—absorbance of the slope at 593 nm.

#### 2.4.3. 2,2′-Azino-bis(3-ethylbenzothiazoline-6-sulfonic acid) (ABTS) Analysis

The ABTS assay was conducted as described elsewhere [32]. An ABTS radical solution was generally prepared and diluted to obtain an absorbance between 0.680 and 0.720 at 734 nm. Next, 20 μL of the sample and 180 μL of ABTS were placed in a microplate. The blank reagent (deionized water) and the calibration curve were prepared using Trolox (T), with concentrations from 5 to 100 μg/mL, starting from the stock solution with a 100 mg/L concentration. After that, the microplate was read at 734 nm using a BioTek Synergy HT plate reader. The ABTS was then expressed as % of inhibition.

In this method, the % inhibition was obtained using Equation (2).

(2) $${\%}_{inhibition}=\frac{{Abs}_{{ABTS}^{\cdot +}}-{Abs}_{a}}{{Abs}_{{ABTS}^{.+}}}\times 100$$

where

${Abs}_{{ABTS}^{\cdot +}}$—absorbance of the radical ABTS at 734 nm.

${Abs}_{a}$—absorbance of the samples/standard at 734 nm.

#### 2.4.4. Antioxidant Potency Composite Index (APCI) Calculation

An overall antioxidant potency composite index (APCI) was calculated following the methodology of Seeram et al. [33], where an equal weight was assigned to all assays. For each test (FRAP and ABTS), the best-performing sample was assigned an index value of 100, and the index score for the other samples was calculated as follows (Equation (3)):

(3) $${APCI}_{\left(FRAP\;or\;ABTS\right)}=\frac{Sample\;score}{Best\;score}\times 100$$

The APCI for a specific extract was determined as the average of the index scores across all tests [33].

### 2.5. Greenness Evaluation

The greenness of the extraction techniques was assessed using AGREEprep 0.91 an analytical metric tool for sample preparation. The AGREEprep tool was developed as an analytical metric to assess the environmental sustainability of sample preparation methods based on ten principles of green sample preparation. This tool assigns scores on a scale of 0 to 1 for using safer solvents, waste reduction, minimizing energy and materials, and operator safety. Each criterion is weighted to reflect its relative importance, allowing for flexible and adaptable evaluations. Moreover, AGREEprep presents the results using circular pictograms, making it easy to identify strengths and areas for improvement in each method evaluated [34,35].

Examples of AGREEprep’s application have demonstrated its usefulness in a wide range of analytical methods, helping to identify practices that have less impact on the environment, as well as improvements to increase the overall sustainability of sample preparation methods [34,35].

### 2.6. Statistical Analysis

The data collected were analyzed using inferential analysis, including comparisons among groups and correlation analysis. A two-way ANOVA was carried out on TPC, FRAP, and ABTS by the seaweed extract and method (SWE, UAE, and SLE). Group comparisons were performed using Tukey’s HSD post hoc tests. A Pearson’s correlation analysis was performed to check for a linear correlation and its direction between the values. A two-tailed *p*-value < 0.05 was considered to indicate statistical significance. Statistical analyses were conducted using SPSS software (IBM Corp. Released 2023. IBM SPSS Statistics for Windows, Version 29.0.2.0 Armonk, NY, USA: IBM Corp.) [34,35].

## 3. Results and Discussion

Macroalgae contains many bioactive compounds with antioxidant properties, including polyphenols, carotenoids, flavonoids, and vitamins (such as Vitamins C and E). So, effective extraction methods are essential to obtain these compounds. This section discusses the antioxidant potential of different algae families (brown, green, and red algae). An RSM analysis was performed to estimate the optimal experimental extraction conditions for each seaweed species to reduce the number of assays. Several experimental runs were required to ensure the statistical rigor and reliability of the data, resulting in 17 parameter combinations for the CCD model and 15 for the BBD model.

The BBD and CCD models were used to establish the optimal model for the 10 seaweeds studied using the TPC and FRAP values as responses, which allowed for the most suitable model for the seaweeds to be determined. The interpretation and analysis of the results obtained by the TPC and FRAP methods provided valuable information on the variation of these two values under different extraction conditions for the same algal species.

The experimental data were collected and analyzed using specialized software to obtain the best extraction conditions for each type of seaweed species. The results, ANOVA, and fit statistics are detailed in the Supplementary Materials section.

### 3.1. Brown Seaweed

#### 3.1.1. Experimental Design and SLE for Brown Seaweed

Table 1 presents the TPC and FRAP results for the experimental design and optimization of the extraction conditions using SLE for the brown seaweed analyzed in this study.

The results (Table 1) show that the CCD model was more suitable for the brown species. In addition, it can also be observed that no model proved suitable for some seaweed responses, especially for the FRAP value. Notably, the predicted R^2^ for the TPC response in the seaweed *F. vesiculosus* was low (0.0696). However, this represented the best model obtained compared to the others, and it still showed a difference of less than 2% between the predicted and experimental values.

The ratio factor (the proportion between the algae biomass and the extraction solvent) was important in determining the TPC and FRAP values when the same temperature and time factors were considered. Higher ratios in brown algae, such as 1:75 and 1:100, consistently resulted in higher TPC and FRAP values. This suggests that increasing the ratio of algae biomass to extraction solvent increases the efficiency of extracting phenolic compounds. In the case of brown seaweed, a biomass/solvent ratio of 1:75 resulted in the highest TPC yield.

A higher biomass/solvent ratio enhances the interaction between the solvent and target compounds, resulting in improved solubility and greater extraction efficiency. This finding aligns with studies that have reported that increasing the volume of solvent improves the extraction of phlorotannins [36].

In addition, the temperature factor and the extraction time influenced the TPC and FRAP values. In general, a high temperature (75 °C) and the shortest extraction time (1 h) revealed the highest TPC and FRAP values, suggesting that these species show a rapid release of phenolic compounds and antioxidants at slightly elevated temperatures.

Furthermore, the TPC and FRAP results show few discrepancies between the predicted and experimental values. In general, for both responses, the experimental values were generally lower than the predicted values. For example, for *U. pinnatifida*, the model predicted a TPC value of 4.62 mg GAE/g d.w., while the experimental value was only 2.54 mg GAE/g d.w.. For the same alga, but for the FRAP values, the predicted value was 17.8 mg AA/g d.w., and the experimental value was 16.6 mg AA/g d.w.. These results reinforce the reliability of the model for brown algae, indicating that the model was more effective at predicting the antioxidant response than the phenolic one, with a difference of less than 10%.

#### 3.1.2. Analysis of the TPC and Antioxidant Capacity for Brown Seaweed

**Figure 3:**
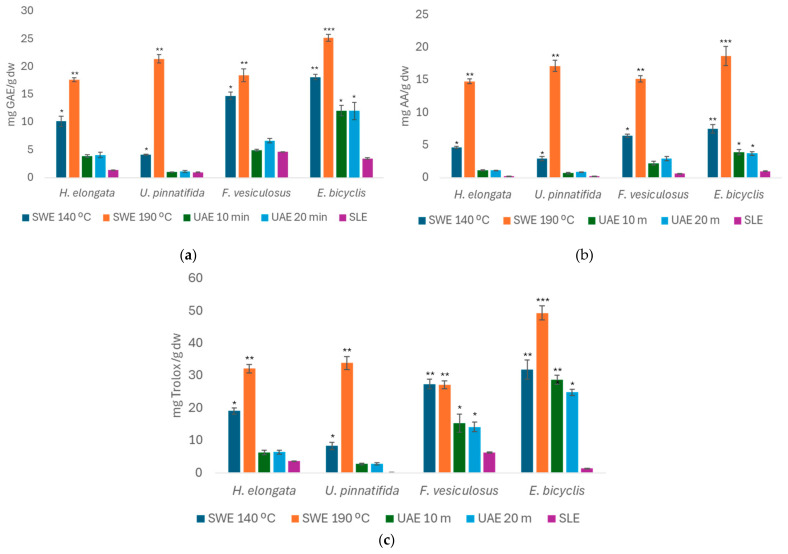
Total phenolic content (TPC, (**a**)), ferric-reducing antioxidant power (FRAP, (**b**)), and ABTS radical-scavenging activity (**c**) values obtained for brown seaweed and different extraction methods. Bars represent mean values, with significance indicated by *,** and *** for *p* < 0.05, denoting extracts significantly different from others within each species.

Figure 3 shows the TPC, FRAP, and ABTS values of the brown seaweeds, expressed in mg of GAE/g d.w., mg AA/g d.w., and mg Trolox/g d.w., respectively, for the extracts obtained from the SLE (optimized conditions), SWE, and UAE.

According to Figure 3a, the brown seaweed analyzed showed the highest TPC values in the SWE extraction at 190 °C compared to the other extractions. The SLE method generally gave values of less than 2.00 mg GAE/g d.w..

For the SWE method, the seaweed *E. bicyclis* stands out, with higher values at 190 °C, around 25.2 ± 0.595 mg GAE/g d.w., followed by the SWE at 140 °C, with a value of 18.1 ± 0.468 mg GAE/g d.w.. The seaweeds *U. pinnatifida* and *F. vesiculosus* also showed higher TPC values for the SWE method at 190 °C, obtaining around 21.4 ± 0.760 mg GAE/g d.w. and 18.4 ± 1.13 mg GAE/g d.w., respectively. The UAE method shows values of less than 5.00 mg GAE/g d.w. for all of the brown seaweeds, except for *E. bicyclis*, where the value is approximately 12.0 mg GAE/g d.w. for both methods. Finally, all seaweeds showed lower TPC values with the SLE method compared to the other methods.

Sapatinha et al. [37] evaluated the biological activities of extracts from two red algae (*Porphyra* sp. and *Gracilaria gracilis*) and two brown algae (*Alaria esculenta* and *Saccharina latissima*) using different extraction methods (enzymatic methods and methods assisted by ball milling and hot water). Focusing on the brown algae, the phenolic compounds showed a variety, with *A. esculenta* ranging from 32.12 ± 0.51 to 68.43 ± 0.22 mg GAE/g d.w. and *S. latissima* ranging from 32.12 ± 0.51 to 104.69 ± 2.47 mg GAE/g d.w..

Sumampouw et al. [38] investigated the optimization of phenolic antioxidant extraction from *F. vesiculosus* using pressurized liquid extraction. Their findings of higher TPC values at higher temperatures and shorter extraction times support the effectiveness of elevated temperatures and shorter durations in extracting bioactive compounds [38].

The analysis of the FRAP values, shown in Figure 3b, reveals considerable variation between species and between the extraction methods used.

The highest FRAP values are observed in the brown seaweeds *H. elongata* and *E. bicyclis*, especially in the extracts obtained by SWE at 190 °C, with around 14.8 ± 0.4 and 18.7 ± 1.5 mg AA/g d.w., respectively. Moreover, regardless of the seaweed species, the SLE method showed lower values than the other extraction methods (SWE and UAE), highlighting its limited efficiency in extracting antioxidant compounds.

In addition, the UAE method also produced high results for *E. bicyclis*, with values close to those obtained by SWE. This can be attributed to the fact that ultrasound facilitates the rupture of seaweed cells, which efficiently releases antioxidant compounds, especially at longer extraction times (UAE for 20 min) [39]. Thus, it can be concluded that these seaweed extracts effectively neutralize free radicals and can, therefore, be considered effective in protecting against oxidative stress.

The FRAP value of methanolic extracts of brown seaweed (*Ascophyllum nodosum*, *Laminaria hyperborea*, *Pelvetia canaliculata*, *F. vesiculosus*, and *Fucus serratus*) was analyzed in a recent study [40]. Seaweed was mixed with methanol and distilled water and incubated at 40 °C in the dark for 3 h, and the methanol was removed by evaporation. The results showed a range between 109.8 and 25.6 μM AA/g d.w.. Compared with the data from this study, only the seaweed *E. bicyclis* is within this range, while the other seaweeds showed lower values for all extraction methods, except for the SWE at 190 °C.

In turn, Ummat et al. [41], also analyzed the antioxidant capacity of seven brown seaweeds (*F. serratus*, *F. vesiculosus*, *Fucus spiralis*, *H. elongata*, *Halidrys siliquosa*, *Laminaria digitata*, *Laminaria saccharina*, *Laminaria hyperborea*, *A. nodosum*, *Alaria esculenta,* and *P. caniculata*) using Trolox as a standard and analyzed the extracts obtained by UAE with 50% ethanol. The FRAP results obtained by the UAE method ranged from 63.9 ± 0.74 mg Trolox/g to 7.8 ± 0.30 mg Trolox/g. It was concluded that the UAE method significantly increased the antioxidant capacity compared to the conventional extraction (SLE), with *F. serratus* as the species with the most significant antioxidant potential. However, this was not observed in the current study, as the UAE showed relatively lower FRAP values, especially at shorter times. Furthermore, although brown seaweeds have higher FRAP values, the values obtained are outside the range presented in the article [41].

Finally, antioxidant activity using ABST is presented in Figure 3c, in which brown seaweed showed an interesting trend, being the best performer in terms of antioxidant capacity overall, with SWE emerging as the most effective method. For the macroalgae *F. vesiculosus,* the SWE showed no significant difference between the two extraction temperatures, obtaining values of around 27.0 mg Trolox/g d.w.. For other seaweeds, such as *H. elongata*, *U. pinnatifida*, and *E. bicyclis*, the highest values were observed for SWE at 190 °C, with 32.27 ± 1.3, 33.9 ± 2.0, and 49.4 ± 2.2 mg Trolox/g d.w., respectively. Finally, for *E. bicyclis*, the SWE at 140 °C showed the highest ABTS value, reaching around 32.0 ± 3.0 mg Trolox/g d.w.. The SLE method exhibited lower values compared to the other extraction methods.

Although the UAE methods presented lower values, they demonstrated a reasonable extraction of antioxidants, as observed for *E. bicyclis*. Values of 28.8 ± 1.4 mg Trolox/g d.w. for UAE at 10 min and 24.9 ±0.9 mg Trolox/g d.w. for UAE at 20 min were obtained. For the remaining seaweed, the ABTS values for the extracts obtained by this method do not exceed the value of approximately 6.00 mg/g of extract, except for the alga *F. vesiculosus*, which reaches around 15.0 mg Trolox/g d.w. for both conditions.

McDonnell et al. [42] investigated the antioxidant capacities of 11 species of brown seaweed collected on the west coast of Ireland using the ABTS, DPPH, and TEAC assays. For the ABTS assay, the results varied between 3.33 ± 0.18 mg Trolox/g d.w. and 114.19 ± 1.03 mg Trolox/g d.w., with the ethanolic extract of *H. siliquosa* showing significantly higher antioxidant capacity than that of the other species. Also, in this study, it is important to note that the results of the ABTS values for the seaweed *H. elongata* showed a higher value (50.06 mg Trolox/g d.w.) compared to our study (30 mg Trolox/g d.w.) [42]. The higher ABTS value can be attributed to differences in the method of extracting antioxidant compounds and the choice of solvents. McDonnell et al. [42] employed extraction assisted by mechanical agitation and centrifugation with 50% ethanol as the solvent.

Subbiah et al. [43] analyzed five species of brown macroalgae (*Cystophora* sp., *Ecklonia radiata*, *Durvillaea* sp., *Phyllospora comosa*, and *Sargassum* sp.), evaluating their antioxidant capacity using the ABTS assay, among others. The ABTS values varied considerably between the freeze-dried samples, ranging from 19.61 ± 1.58 mg AAE/g (*P. comosa*) to 47.74 ± 4.02 mg AAE/g (*Cystophora* sp.).

The values obtained are within the range of the results obtained in the literature [43], although the extraction method, solvent, and standard solution are different. In this article, extraction by maceration with 70% ethanol added to 0.1% formic acid and ascorbic acid as a standard was used.

### 3.2. Red Seaweed

#### 3.2.1. Experimental Design and SLE for Red Seaweed

Table 2 presents the TPC and FRAP results for the experimental design and optimization of the extraction conditions using SLE for the red seaweed analyzed in this study.

Table 2 shows that the BBD model was most suitable for the red species, except for the algae *C. crispus*. It can also be seen that no model was suitable for the seaweed *G. gracilis* and *P. dioica* using the FRAP and TPC responses, respectively. For the seaweed *P. palmata*, the CCD model does not show a significant fit for any of the responses analyzed (FRAP and TPC), indicating that the experimental conditions and/or variability in the data do not support accurate modeling.

In addition, a low predicted R^2^ (0.0682) was obtained for the TPC response in *C. crispus*. However, this represented the best model obtained compared to the others, despite presenting a difference of 44.7% between the predicted and experimental values.

Regardless of the model chosen, the ratio factor was 1:75, and the extraction time was 3 h, which resulted in high TPC and FRAP values. The temperature ranged from 69 to 75 °C, except for *P. palmata*, which had a low temperature (25 °C), indicating that room temperature improves the extraction of phenolic compounds and antioxidants in this species. The *G. gracilis* algae had an optimum extraction time of 1.51 h, much lower than the other red algae.

This result is in line with studies that have reported that, for red algae, increasing the volume of solvent and using moderate temperatures favors the extraction of phenolic compounds, such as flavonoids and phenolic acids [44,45].

However, the TPC and FRAP results show few discrepancies between the predicted and experimental values, except for the alga *C. crispus*. For both responses, the experimental values were generally lower than the predicted values. For *C. crispus*, the model predicted a TPC value of 3.80 mg GAE/g d.w., while the experimental value was only 2.10 mg GAE/g d.w.. For the same seaweed, but for the FRAP values, the predicted value was 10.4 mg AA/g d.w., and the experimental value was 7.19 mg AA/g d.w.. For the TPC and FRAP response, this alga showed differences of 44.7 and 30.9%, respectively. However, a difference of less than 15% was obtained for the other algae, so these results reinforce the model’s reliability for red algae, indicating that the model was more effective in predicting the antioxidant and phenolic response.

#### 3.2.2. Analysis of the TPC and Antioxidant Capacity for Red Seaweed

**Figure 4:**
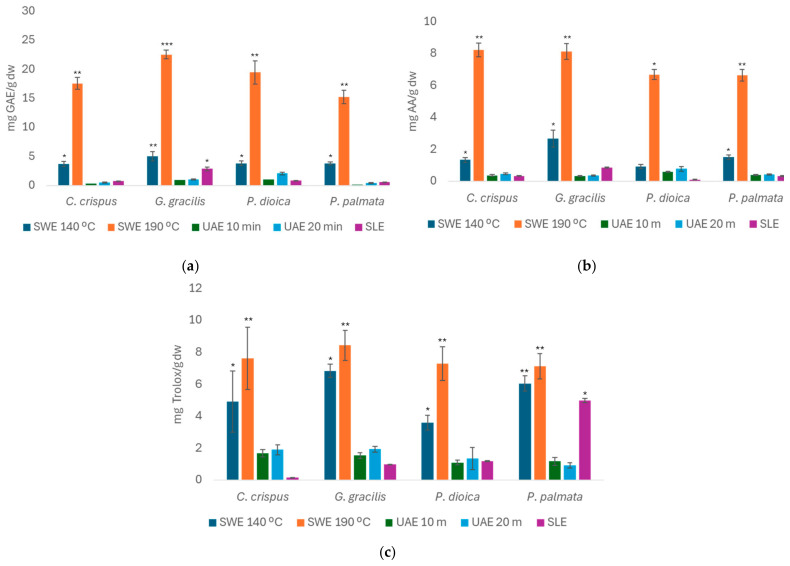
Total phenolic content (TPC, (**a**)), ferric-reducing antioxidant power (FRAP, (**b**)), and ABTS radical-scavenging activity (**c**) values obtained for red seaweed and different extraction methods. Bars represent mean values, with significance indicated by *,** and *** for *p* < 0.05, denoting extracts significantly different from others within each species.

Figure 4 shows the TPC, FRAP, and ABTS values of the red seaweeds, expressed as mg of GAE/g d.w., mg AA/g d.w., and mg Trolox/g d.w., respectively, for the extracts obtained from the SLE (optimized conditions), SWE, and UAE.

Referring to Figure 4a, *G. gracilis* stood out in the SWE method at 190 °C, with values close to 23 mg GAE/g d.w., followed by *P. dioica* (19.5 ± 2.03 mg GAE/g d.w.), *C. crispus* (17.6 ± 1.02 mg GAE/g d.w.), and *P. palmata* (15.2 ± 1.17 mg GAE/g d.w.). The SWE 140 °C method gave values close to 5 mg GAE/g d.w., while the UAE and SLE methods yielded lower values. However, the SLE method shows a slightly higher TPC value for the seaweed *G. gracilis* and also shows higher values compared to the UAE method.

The red seaweed had the highest TPC values at SWE 190 °C, primarily due to the extraction method used. In line with this, a study [46] using SWE on different seaweeds found that the antioxidant capacity of the extracts obtained at 200 °C was significantly higher than those extracted at 100 °C, confirming the values in this study, since SWE at 190 °C is generally the most efficient method for extracting phenolic compounds in seaweeds. In addition, this study also observed the formation of antioxidants during the SWE process in different seaweeds. The increase in antioxidant capacity was attributed to the production of new compounds resulting from Maillard reactions and caramelization during heat treatment. It is worth noting that all of the extracts obtained by SWE at 190 °C had a noticeable toasted aroma [46].

Similar to the TPC values, red seaweed showed higher FRAP values (Figure 4b) for the SWE method at 190 °C in the species evaluated, with *C. crispus* and *G. gracilis* standing out with values of 8.22 ± 0.43 and 8.13 ± 0.48 mg AA/g d.w., respectively.

In another study, Khatulistiani et al. [47] studied the bioactivities of extracts of Indonesian red seaweeds (*Eucheuma* sp., *Gelidium* sp., *Eucheuma spinosum*, *Halymenia* sp., and *Rhodopeltis* sp.). These seaweed bioactive compounds were extracted by maceration with distilled water (ratio 1:2, *w*/*v*) for 24 h at cold temperatures. The FRAP values ranged from 15.81 ± 0.57 to 19.08 ± 0.66 AA mg/g. It should be noted that these values are much higher than the FRAP values obtained in the current study. The extraction method can explain this difference, although the SWE method at 190 °C for red seaweed showed the highest FRAP values [47].

Finally, red algae’s free-radical-scavenging activity was analyzed by ABTS for the different extraction methods, as demonstrated in Figure 4c. In general, they have a lower antioxidant capacity than the other algae groups, with values of around 8.00 mg Trolox/g d.w.. However, compared to the other groups, the SWE seems to be more efficient at extracting antioxidant compounds at 190 °C, as observed for the seaweeds *G. gracilis* (8.43 ± 0.95 mg Trolox/g d.w.) and *C. crispus* (7.63 ± 1.95 mg Trolox/g d.w.). The UAE method, on the other hand, showed even lower values than the other seaweed groups, with a maximum of 2.00 mg/g d.w.. Furthermore, the SLE method performs similarly to UAE, although with generally lower values, except for the alga *P. palmaria*, which reached 4.98 ± 0.13 mg Trolox/g d.w..

Pimentel et al. [48] evaluated the antioxidant capacity of the macroalga *P. dioica*. This macroalgae showed ABTS values of 112.0 ± 8.8 mg TE/g d.w. and 311.2 ± 39.3 mg TE/g d.w., for the different extraction methods, such as protein isolation based on alkaline solubilization and isoelectric precipitation and sequential enzymatic hydrolysis, respectively. These values are significantly higher than those reported in this study, possibly due to the differences in the extraction methods used.

### 3.3. Green Seaweed

#### 3.3.1. Experimental Design and SLE for Green Seaweed

Table 3 presents the TPC and FRAP results for the experimental design and optimization of the extraction conditions using SLE for the green seaweed analyzed in this study.

Table 3 shows that the BBD and CCD models were chosen for the algae *C. tomentosum* and *U. lactuca*, respectively. Furthermore, the *C. tomentosum* algae has a low predicted R^2^ (0.0682) for the TPC response. However, it represented the best model obtained compared to the others, despite showing a difference of 31.4% between the predicted and experimental values.

Regardless of the model chosen, an optimal temperature of 75 °C was obtained, but the extraction time varied between 1 h (*C. tomentosum*) and 5 h (*U. lactuca*). In addition, higher ratios, such as 1:75 (*C. tomentosum*) and 1:100 (*U. lactuca*), consistently resulted in higher TPC and FRAP values. This suggests that increasing the ratio of seaweed biomass to extraction solvent increases the extraction efficiency of phenolic compounds and antioxidants. A higher biomass/solvent ratio facilitates better contact between the solvent and the target compounds, leading to better solubility and extraction efficiency.

However, the FRAP results show few discrepancies between the predicted and experimental values compared to the TPC results. For both responses, the experimental values were generally lower than the predicted values. For the FRAP response in the algae *C. tomentosum* and *U. lactuca*, differences of 1.33% and 2.91% were obtained, respectively. For the TPC response, differences of 31.4% and 50.9% were obtained for *C. tomentosum* and *U. lactuca*, respectively. These results reinforce the model’s reliability for green algae, indicating that the model was more effective in predicting the antioxidant response than the phenolic response.

#### 3.3.2. Analysis of the TPC and Antioxidant Capacity for Green Seaweed

**Figure 5:**
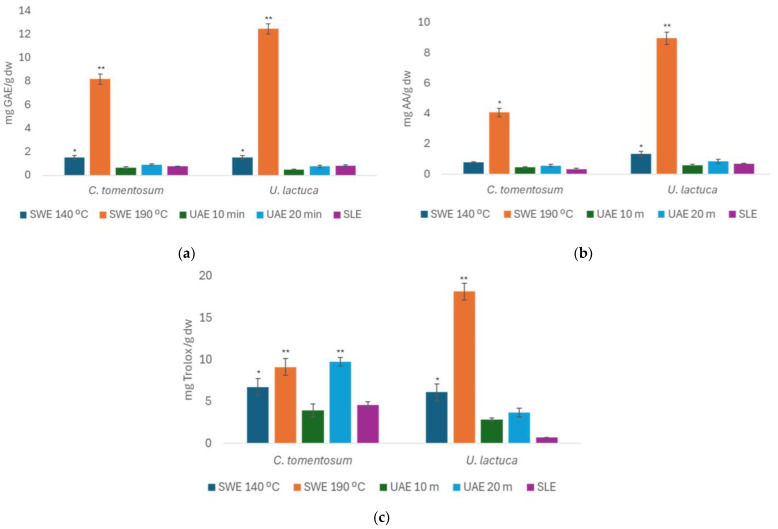
Total phenolic content (TPC, (**a**)), ferric-reducing antioxidant power (FRAP, (**b**)), and ABTS radical-scavenging activity (**c**) values obtained for green seaweed and different extraction methods. Bars represent mean values, with significance indicated by * and ** for *p* < 0.05, denoting extracts significantly different from others within each species.

Figure 5 shows the TPC, FRAP, and ABTS values of the green seaweeds, expressed in mg of GAE/g d.w., mg AA/g d.w., and mg Trolox/g d.w., respectively, for the extracts obtained from the SLE (optimized conditions), SWE, and UAE.

Based on Figure 5a, the green seaweed showed the highest TPC values for the SWE at 190 °C, achieving 5.62 ± 1.40 mg GAE/g d.w. for *U. lactuca*, followed by *C. tomentosum* with 4.44 ± 1.22 mg GAE/g d.w.. The TPC values for the other methods were below 2.00 mg GAE/g d.w.. Additionally, the SLE method yielded values comparable to those obtained with the UAE (20 min) method.

In contrast, a study reported a significantly higher TPC in the *U. lactuca* extracts obtained using acetone as a solvent, with a value of 64.68 ± 1.55 mg GAE/g, compared to the lower values observed in methanolic (51.08 ± 0.72) and ethanolic (58.47 ± 0.54) extracts [49].

Khan et al. [50] investigated various separate drying methods (oven drying, freeze-drying, and sun drying) to quantify the total phenolic content (TPC) and total flavonoid content (TFC) of *Ulva intestinalis* and *Padina tetrastromatica*. The TPC was determined using gallic acid standards, and freeze drying resulted in higher TPC values, with 12.59 ± 1.07 mg GA/g d.w. in *U. intestinalis* and 68.74 ± 1.95 mg GA/g d.w. in *P. tetrastromatica*.

Regarding the FRAP assay, as shown in Figure 5b, *U. lactuca* exhibited the highest antioxidant activity with the SWE at 190 °C, followed by *C. tomentosum*, which also showed significant value under the same conditions. However, *U. lactuca* displayed consistently low values across all other methods.

Gunathilake et al. [51] evaluated the antioxidant activity of the seaweed *Ulva australis* extracted using water, 70% *v*/*v* ethanol, 70% *v*/*v* acetone, and 70% *v*/*v* methanol. The study reported FRAP values ranging from 30 mg AA/g d.w. for the ethanolic extract to 10 mg AA/g d.w. for the aqueous extract. Compared to the results of the present study, *C. tomentosum* exhibited a similar FRAP value for the aqueous extract, regardless of the extraction method. In contrast, *U. lactuca* demonstrated a higher FRAP value, particularly with the SWE at 190 °C.

Finally, as shown in Figure 5c, the ABTS values indicate that green algae generally exhibit lower antioxidant activity than brown algae but perform relatively better than red algae. *U. lactuca* showed the highest antioxidant activity with SWE at 190 °C, achieving 18.2 ± 2.5 mg Trolox/g d.w.. For *C. tomentosum*, the SWE at 190 °C and the UAE at 20 min yielded the highest ABTS values, approximately 10.0 mg Trolox/g d.w.. Additionally, the SLE method produced results for *C. tomentosum* similar to those obtained with the UAE at 10 min.

A previous study evaluated the antioxidant capacity of *U. lactuca* and reported an ABTS value of 89.77 μg TE/g d.w., which aligns with the SLE extraction values observed in this study, as both used the same extraction method. However, the other extraction techniques in this study yielded higher ABTS values [52].

### 3.4. Antioxidant Potency Composite Index (APCI)

Table 4 presents the antioxidant activities of seaweed extracts, as evaluated by two assays: FRAP and ABTS. The results are expressed as antioxidant index scores (%) to normalize and compare the performances across species and methods. The antioxidant potency composite index (APCI) was calculated to integrate the results of both assays, providing a holistic measure of each extract’s overall antioxidant capacity.

Each extraction method (SWE 140 °C, SWE 190 °C, UAE 10 min, UAE 20 min, and SLE) shows varying efficiencies in extracting antioxidant compounds from different seaweed species. SWE at 190 °C demonstrated the highest scores within most species, while other methods like UAE and SLE gave comparatively lower antioxidant capacities.

The antioxidant index scores and APCI values are important for assessing how effectively different extraction techniques can recover bioactive compounds with antioxidant properties. The results suggest that SWE at 190 °C is a superior method for obtaining antioxidant-rich seaweed extracts, as evidenced by the highest FRAP and ABTS scores in several species. For example, *E. bicyclis* shows an APCI score of 46.27%, far surpassing other species and indicating its potent antioxidant activity when processed with SWE at 190 °C.

This analysis emphasizes the importance of selecting the right extraction technique and processing conditions to maximize the recovery of bioactive compounds. Seaweed species also exhibit variability in antioxidant potential, which may be attributed to differences in their biochemical composition [42]. These findings are critical for industries focused on developing functional foods, nutraceuticals, or natural antioxidants, as they highlight the best-performing species and the most effective extraction methods [53].

### 3.5. Correlations Between Methods

Table 5 presents the Pearson correlation coefficients (r) between TPC, FRAP, and ABTS for all seaweed extracts. The correlation values range from −1 to 1, where values closer to 1 indicate a strong positive correlation, 0 indicates no correlation, and values closer to −1 indicate a strong negative correlation.

The strong positive correlations observed between TPC, FRAP, and ABTS suggest that phenolic compounds in the extracts significantly contribute to their antioxidant activity, as measured by both the FRAP and ABTS assays. This supports the well-documented role of phenolics as essential antioxidants in various biological and chemical systems [54].

These results highlight the reliability of TPC as a predictor of antioxidant capacity in seaweed extracts, providing a helpful screening tool for identifying potent antioxidant sources [55]. Furthermore, the high correlation between FRAP and ABTS (r = 0.838) supports the complementary nature of these assays in evaluating antioxidant activity, as they measure different mechanisms of action (electron transfer for FRAP and radical scavenging for ABTS) [56]. Belhadj et al. [55] evaluated the relationship between TPC and antioxidant activities (DPPH, ABTS, and FRAP) in the extracts of the invasive red seaweed *R. okamurae* (E.Y. Dawson) I.K. Hwang, W.J. Lee, and H.S. Kim, 2009. TPC demonstrated a strong positive correlation with the DPPH and FRAP assays (r = 1.000), indicating a similar predictive capacity for antioxidant activity in these seaweed extracts. These findings suggest that phenolic compounds are the primary contributors to the antioxidant capacities of these extracts. In contrast, these authors reported that ABTS did not correlate significantly with TPC, DPPH, or FRAP (*p* > 0.05) [55].

This information is essential for researchers and industries focusing on natural antioxidants, as it demonstrates the consistency across different methods and highlights the role of phenolic content in antioxidant potential.

#### Evaluation of the Extraction Method Greenness (AGREEprep)

**Figure 6:**
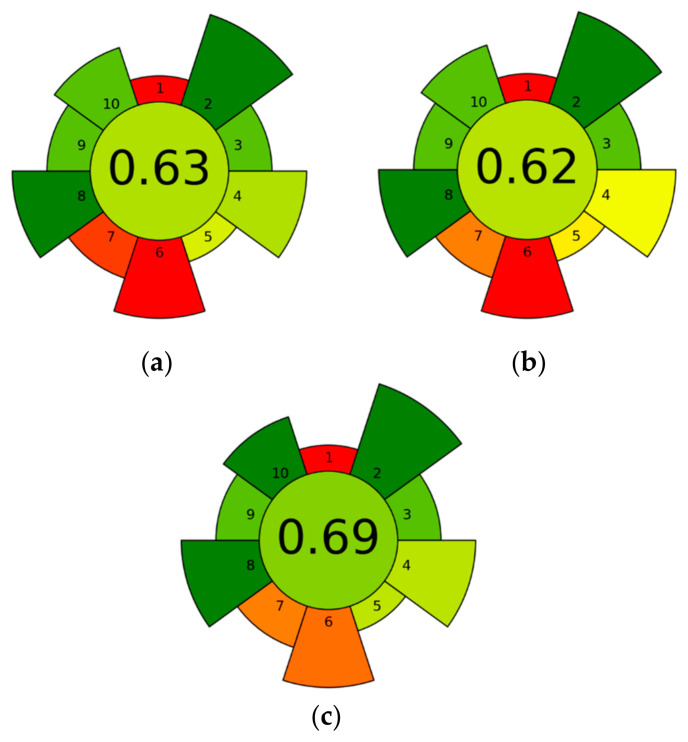
AGREEprep evaluation of the greenness of sample preparation methodologies (SLE (**a**), SWE (**b**), UAE (**c**)), presented as a numerical score (center) and visual representation of compliance with green chemistry principles across 10 criteria.

The greenness of the sample preparation methodologies was assessed using the AGREEprep analytical metric tool (Figure 6), which evaluates compliance with green chemistry principles. AGREEprep assigns a numerical score, ranging from zero (least green) to one (most green), based on 10 criteria that cover various aspects of environmental, safety, and sustainability considerations in sample preparation [34,35].

The AGREEprep evaluation stresses that UAE is the most sustainable method among the three, achieving the highest score (0.69). This result is attributed to its efficient use of energy, minimal waste generation, and operational safety, which align well with the principles of green chemistry [57]. UAE’s performance reflects its suitability for environmentally sensitive applications, particularly in natural product extraction and functional food development, where scalability and sustainability are paramount [57].

While SWE (0.62) showed promise due to its use of water and renewable materials, its energy-intensive nature during subcritical conditions limits its green potential. By integrating heat recovery systems and optimizing pressure and temperature settings, SWE could emerge as a more competitive method in terms of greenness [58]. Its capacity to process larger sample sizes compared to UAE gives it a unique advantage in applications requiring bulk extraction, provided its energy requirements can be mitigated.

SLE (0.63), though relatively traditional, achieved a comparable score to SWE, demonstrating that simple methods can still align moderately with green chemistry principles when using water as the solvent [59]. However, SLE’s inefficiencies in automation, throughput, and waste management suggest that it is best suited for small-scale or preliminary extractions rather than high-throughput industrial processes. Sample-handling and process automation enhancement could improve its performance and broaden its application scope [59].

This analysis highlights the importance of designing extraction methodologies for specific applications while considering their environmental impact. The AGREEprep tool provides a comprehensive framework for evaluating and comparing techniques, allowing researchers to identify greener alternatives and prioritize improvements in energy use, waste management, and process efficiency.

In conclusion, UAE stands out as the most sustainable choice for green extraction, while SWE and SLE present opportunities for optimization. The findings emphasize the potential for all three methods to achieve greater sustainability through targeted innovations, improving the broader goal of integrating green chemistry principles into sample preparation methodologies.

## 4. Conclusions

This study optimized extraction conditions to maximize antioxidant recovery from ten commercially available seaweed species in Portugal, focusing on temperature, biomass-to-solvent ratio, and time. Statistical models (BBD and CCD) were used to optimize the conditions for SLE, and the results were applied to SWE and UAE to evaluate their efficiency. The extraction techniques were assessed for their ability to recover total phenolic compounds (TPC) and antioxidant capacity using FRAP and ABTS assays.

The results identified a biomass-to-solvent ratio of 1:75 and a temperature of 75 °C as optimal for most species, though species-specific variations emphasized the need for tailored optimization. SWE at 190 °C proved to be the most efficient technique for extracting phenolic compounds and antioxidants, particularly in brown algae like *E. bicyclis* and *F. vesiculosus*. Notably, red algae, such as *G. gracilis*, also exhibited a strong antioxidant potential.

Despite the promising results, discrepancies between the predicted and experimental values highlight areas for model refinement. Future research should explore additional antioxidant assays and incorporate sustainability metrics, such as energy efficiency and environmental impact, to enhance extraction processes further.

These findings contribute to developing sustainable, effective extraction methods for producing antioxidant- and phenolic-rich products, supporting their application in functional foods, nutraceuticals, and other industries.

## Figures and Tables

**Table 1:** Response surface methodology (RSM) analysis results for brown seaweeds, showing the evaluation of experimental factors and their influence on the response variables (TPC and FRAP) for optimization of the extraction conditions.

Seaweed		*E. bicyclis*		*F. vesiculosus*		*H. elongata*		*U. pinnatifida*	
**Temperature (°C)**		75		75		55.2		75	
**Ratio (g/mL)**		1:75		1:75		1:74		1:100	
**Time (h)**		1		1		3.5		1	
**Best model**		CCD		CCD		CCD		CCD	
**Response**		TPC (mg GAE/g d.w.)	FRAP (mg AA/g d.w.)	TPC (mg GAE/g d.w.)	FRAP (mg AA/g d.w.)	TPC (mg GAE/g d.w.)	FRAP (mg AA/g d.w.)	TPC (mg GAE/g d.w.)	FRAP (mg AA/g d.w.)
**Model**		Quadratic	N.A	Reduced Cubic	N.A	N.A	Reduced Cubic	Cubic	Reduced Cubic
**Parameters**	**Model (*p*-value)**	0.0002	Null	0.0338	Null	Null	0.0004	0.0028	0.0111
	**Lack-of-fit**	0.142		0.350			0.0630	0.994	0.326
	**(*p*-value) R^2^**	0.971		0.689			0.960	0.996	0.854
	**Adjusted R^2^**	0.933		0.502			0.908	0.981	0.707
	**Predicted R^2^**	0.783		0.0696			0.706	0.994	0.437
	**Adeq precision**	15.5		7.51			15.9	28.9	9.35
**Equation**		(a)	-	(b)	-	-	(c)	-	(d)
**Predicted Value**		19.1	-	45.1	-	-	54.5	4.62	17.8
**Real Value**		21.1	195	44.4	178	9.98	36.5	2.54	16.6

N.A—No model proved suitable for the seaweed regarding that response. (a) TPC = −1.62 + 0.253 × A + 0.452 × B − 3.369 × C + 0.00155 × A × B − 0.00427 × A × C − 0.00251 × B × C − 0.00256 × A^2^ − 0.00413 × B^2^ + 0.594 × C^2^; (b) TPC = 45.8 − 1.51 × A + 0.158 × B − 11.1 × C + 0.583 × A × C + 0.0185 × A^2^ − 0.00643 × A^2^ × C; (c) FRAP = 15.1 − 0.234 × A + 1.05 × B − 3.42 × C − 0.0279 × A × B + 0.165 × A × C + 0.0153 × A^2^ − 0.0171 × B^2^ − 0.000346 × A^2^ × B + 0.000596 × A × B^2^; (d) FRAP = −143 + 1.95 × A + 4.79 × B + 1.37 × C − 0.0765A × B + 0.0155 × A^2^ − 0.0328 × B^2^ − 0.000138 × A^2^ × B + 0.000569 × A × B^2^.

**Table 2:** Response surface methodology (RSM) analysis results for red seaweeds, showing the evaluation of experimental factors and their influence on the response variables (TPC and FRAP) for optimization of the extraction conditions.

Seaweed		*C. crispus*		*G. gracilis*		*P. palmata*		*P. dioica*	
**Temperature (°C)**		69		73		25		75	
**Ratio (g/mL)**		1:75		1:75		1:75		1:75	
**Time (h)**		3		1.51		3		3	
**Best model**		CCD		BBD		BBD		BBD	
**Response**		TPC (mg GAE/g d.w.)	FRAP (mg AA/g d.w.)	TPC (mg GAE/g d.w.)	FRAP (mg AA/g d.w.)	TPC (mg GAE/g d.w.)	FRAP (mg AA/g d.w.)	TPC (mg GAE/g d.w.)	FRAP (mg AA/g d.w.)
**Model**		Quadratic	Reduced Quadratic	Quadratic	N.A	N.A	N.A	N.A	Linear
**Parameters**	**Model (*p*-value)**	0.0123	0.0004	0.0019	Null	Null	Null	Null	0.0235
	**Lack-of-fit**	0.206	0.333	0.0952					0.719
	**(*p*-value) R2**	0.889	0.675	0.974					0.563
	**Adjusted R2**	0.747	0.629	0.928					0.444
	**Predicted R2**	0.0682	0.540	0.610					0.172
	**Adeq precision**	10.5	7.33	14.6					7.01
**Equation**		(a)	(b)	(c)	-	-	-	-	(d)
**Predicted Value**		3.80	10.4	3.36	-	-	-	-	5.47
**Real Value**		2.10	7.19	2.87	13.3	1.10	4.78	1.17	4.68

N.A—No model proved suitable for the seaweed regarding that response; (a) TPC = 9.36 + 0.148 × A − 0.214 × B + 0.0978 × C − 0.000737 × A × B − 0.00328 × A × C + 0.00659 × B × C − 0.000529 × A^2^ + 0.00105 × B^2^ − 0.0515 × C^2^; (b) FRAP = 30.8 − 0.612 × A + 0.00459 × A^2^; (c) TPC = 0.0644 + 0.0115 × A + 0.0421 × B + 0.344 × C + 0.000388 × A × B − 0.0034 × A × C + 0.00055 × B × C − 0.000227 × A^2^ − 0.000311 × B^2^ − 0.0317 × C^2^; (d) FRAP =16.52 − 0.1313 × A − 8.0.07677 × B − 0.2182 × B^2^.

**Table 3:** Response surface methodology (RSM) analysis results for green seaweeds, showing the evaluation of experimental factors and their influence on the response variables (TPC and FRAP) for optimization of the extraction conditions.

Seaweed		*C. tomentosum*		*U. lactuca*	
**A-Temperature (°C)**		75		75	
**B-Ratio (g/mL)**		1:75		1:100	
**C-Time (h)**		1		5	
**Best model**		BBD		CCD	
**Response**		TPC (mg GAE/g d.w.)	FRAP (mg AA/g d.w.)	TPC (mg GAE/g d.w.)	FRAP (mg AA/g d.w.)
**Model**		Reduced Quadratic	Quadratic	Reduced Cubic	Quadratic
**Parameters**	**Model (** ***p*** **-value)**	0.0123	0.0002	0.0012	0.0063
	**Lack-of-fit**	0.206	0.520	0.5297	0.614
	**(*p*-value) R^2^**	0.889	0.990	0.756	0.910
	**Adjusted R^2^**	0.747	0.972	0.674	0.794
	**Predicted R^2^**	0.0682	0.893	0.466	0.370
	**Adeq precision**	10.5	26.9	9.85	11.4
**Equation**		(a)	(b)	(c)	(d)
**Predicted Value**		3.22	15.0	4.77	10.3
**Real value**		2.21	14.8	2.35	10.6

(a) TPC = −6.36 + 0.0523 × A + 0.155 × B + 0.819 × C − 0.0135 × A × C − 0.00103 × B^2^; (b) FRAP = 34.4 − 0.878 × A − 0.250 × B − 1.56 × C + 0.00301 × A × B + 0.00525 × A × C + 0.0173 × B × C + 0.007490 × A^2^ + 0.00106 × B^2^ + 0.0579 × C^2^; (c) TPC = 4.10 + −0.204 × A + 0.02246104 × B + 0.393536 × C + 0.00209 × A^2^; (d) FRAP = 7.24 + 0.0710 × A −0.116 × B − 1.25 × C + 0.00121 × A × B + 0.0275 × A × C − 0.0157 × B × C − 0.00221 × A^2^ + 0.00114 × B^2^ + 0.204 × C^2^.

**Table 4:** Antioxidant index scores for FRAP and ABTS assays and the antioxidant potency composite index (APCI) for different seaweed extracts were obtained using various extraction methods. The table highlights differences in antioxidant capacity among species and extraction methodologies.

		*C. crispus*	*C. tomentosum*	*E. bicyclis*	*F. vesiculosus*	*G. gracilis*	*H. elongata*	*P. dioica*	*P. palmata*	*U. lactuca*	*U. pinnatifida*
**FRAP index (%)**	**SWE 140 °C**	7.19	4.29	40.1	34.4	14.4	24.7	4.92	8.07	7.13	15.6
	**SWE 190 °C**	44.1	21.9	100	81.4	43.5	79.2	35.8	35.6	48.1	91.9
	**UAE 10 min**	2.04	2.46	20.9	11.8	1.74	6.01	3.25	2.13	3.20	3.66
	**UAE 20 min**	2.54	3.09	19.9	15.8	1.99	5.87	4.21	2.31	4.57	4.57
	**SLE**	1.84	1.82	5.35	3.42	4.65	1.10	0.63	1.80	3.73	1.20
**ABTS index** **(%)**	**SWE 140 °C**	9.96	13.7	64.7	55.5	13.9	38.8	7.28	12.2	12.4	16.9
	**SWE 190 °C**	15.4	18.5	100	55.2	17.1	65.3	14.8	14.4	36.8	68.7
	**UAE 10 min**	3.39	8.03	58.3	31.1	3.11	12.8	2.23	2.39	5.83	5.59
	**UAE 20 min**	3.86	19.8	50.5	28.8	3.91	13.1	2.75	1.85	7.48	5.66
	**SLE**	0.300	9.32	2.99	12.6	2.02	7.41	2.42	10.1	1.47	0.360
**APCI**		9.06	10.29	46.27	33.02	10.63	25.41	7.82	9.09	13.07	21.42

**Table 5:** Pearson correlation matrix showing the relationship between total phenolic content (TPC), ferric-reducing antioxidant power (FRAP), and ABTS radical-scavenging activity for all seaweed extracts.

	TPC	FRAP	ABTS
TPC	1	0.904	0.797
FRAP	0.904	1	0.838
ABTS	0.797	0.838	1

The correlation is significant at the 0.01 level (2 tailed). The color intensity represents the strength of the correlation, with darker shades indicating higher correlations. r > 0.7 (strong correlation), and r = 1 (perfect correlation).

## Data Availability

The original contributions presented in this study are included in the article/Supplementary Materials. Further inquiries can be directed to the corresponding authors.

## Supplementary Materials

The following supporting information can be downloaded at: https://www.mdpi.com/article/10.3390/foods14030453/s1: Figure S1: Model graph for the factor AB (temperature × biomass:solvent ratio) of ANOVA for Linear model for *C. crispus*, Figure S2: Model graph for the factor AB (temperature × biomass/solvent ratio) of ANOVA for reduced quadratic model for *C. crispus*, Figure S3: Model graph for the factor AB (temperature × biomass/solvent ratio) of ANOVA for quadratic model for *C. crispus*. Table S1: Conditions tested for the CCD model, Table S2: Conditions tested for the BBD model, Table S3: TPC (mg GAE/g dw) and FRAP (mg AA/L) results and the BBD predicted values according to each condition for *C. crispus*, Table S4: ANOVA for Quadratic model for *C. crispus*, Table S5: Constrains of the optimal conditions of ANOVA for Quadratic model for *C. crispus*, Table S6: TPC (mg GAE/g dw) and FRAP (mg AA/L) results and the CCD predicted values according to each condition for *C. crispus*, Table S8: Fit Statistics of ANOVA for Reduced Quadratic model for *C. crispus*, Table S9: ANOVA for Quadratic model for *C. crispus*. Table S10: Fit Statistics of ANOVA for Quadratic model for *C. crispus*, Table S11: Constrains of the optimal conditions of ANOVA for Quadratic model for *C. crispus*
