# Supplementary material for: Optimized Extraction Protocols for Bioactive Antioxidants from Commercial Seaweeds in Portugal: A Comparative Study of Techniques
Source: Foods. 2025 Jan 30;14(3):453. doi: 10.3390/foods14030453 (PMC11816920; doi:10.3390/foods14030453)
Supplement: Supplementary file 1 [file foods-14-00453-s001.zip › foods-3399622-supplementary.pdf]

Supplementary material

A predetermined number of experimental runs were performed to ensure statistical validity and reliability based on the design matrix associated with the chosen factorial design, resulting in 17 parameter combinations for the CCD model (Table S1) and 15 for the BBD model (Table S2).

Table S1. Conditions tested for the CCD model.

Run	Temperature (°C)	Ratio	**Time**
1	25	1:100	1
2	50	1:125	3
3	75	1:150	1
4	50	1:125	3
5	75	1:125	3
6	25	1:150	1
7	25	1:125	3
8	50	1:150	3
9	50	1:125	1
10	25	1:150	5
11	50	1:100	3
12	75	1:100	1
13	75	1:100	5
14	75	1:150	5
15	50	1:125	3
16	25	1:100	5
17	50	1:125	5

Table S2. Conditions tested for the BBD model.

Run	Temperature (°C)	Ratio	**Time**
1	50	1:75	5
2	75	1:25	3
3	50	1:25	1
4	25	1:25	3
5	25	1:50	5
6	75	1:50	5
7	50	1:25	5
8	50	1:50	3
9	50	1:75	1
10	50	1:50	3
11	75	1:50	1
12	50	1:50	3
13	25	1:50	1
14	75	1:75	3
15	25	1:75	3

This section presents an example (*C. crispus*) of the data obtained from the statistical analysis carried out using Design Expert 11 software. This data includes the results of the predetermined number of experimental trials, as well as the corresponding predicted TPC and FRAP values, derived using ANOVA for the best-fit model and Model Graph.

Box-Behnken Design

Table S3. - TPC (mg GAE/g dw) and FRAP (mg AA/L) results and the BBD predicted values according to each condition for C. crispus.

Run	Factor A	Factor B	Factor C	Response FRAP	Response TPC
**1**	50	150	5	8.19	4.47
**2**	75	100	3	7.25	2.60
**3**	25	100	3	10.4	1.09
**4**	75	150	3	9.51	2.52
**5**	25	150	3	27.2	1.52
**6**	50	150	1	10.8	2.08
**7**	50	125	3	10.0	2.86
**8**	25	125	1	12.	0.813
**9**	50	125	3	8.31	2.96
**10**	50	100	5	21.7	3.32
**11**	50	100	1	19.5	1.82
**12**	25	125	5	15.9	1.85
**13**	75	125	1	6.15	1.40
**14**	50	125	3	10.8	2.36
**15**	75	125	5	14.8	3.03

- Response 1: FRAP

The BBD model does not present a significant fit for FRAP responses.

- Response 2: TPC

ANOVA for Quadratic model

Table S4 - ANOVA for Quadratic model for C. crispus.

Source	Sum of Squares	df	Mean Square	F-value	p-value	
**Model**	12.1	9	1.35	11.3	0.0079	significant
**A-Temperature**	2.29	1	2.29	19.1	0.0072	
**B-Ratio**	0.384	1	0.384	3.20	0.134	
**C-Time**	5.39	1	5.39	45.0	0.0011	
**AB**	0.0648	1	0.0648	0.541	0.495	
**AC**	0.0881	1	0.0881	0.736	0.430	
**BC**	0.202	1	0.202	1.69	0.250	
**A^2**	3.47	1	3.47	29.0	0.0030	
**B^2**	0.116	1	0.116	0.972	0.369	
**C^2**	0.000800	1	0.000800	0.00700	0.937	
**Residual**	0.599	5	0.120			
**Lack of Fit**	0.395	3	0.131	1.29	0.464	not significant
**Pure Error**	0.204	2	0.102			
**Cor Total**	12.7	14				

Model Graph

The Model Graph provides a comprehensive visualization of the Box-Behnken Design's linear model for *C. crispus*. Figure S1. showcases the relationship between the TPC values (mg GAE/g dw). and the significant factor AB (temperature × biomass:solvent ratio).

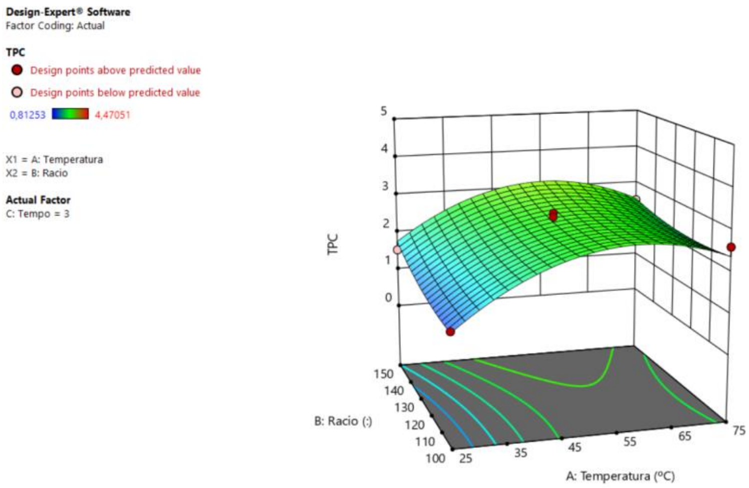

Figure S1- Model graph for the factor AB (temperature × biomass:solvent ratio) of ANOVA for Linear model for C. crispus.

Constrains

Tables S5 to S8 present the constraints to identify the optimal extraction conditions for *C. crispus*. These constraints were systematically applied during the BBD process to ensure that the extraction conditions remained within specified boundaries. thereby allowing us to explore the most favorable combinations of variables.

Table S5 - Constrains of the optimal conditions of ANOVA for Quadratic model for C. crispus.

Name	Goal	Lower Limit	Upper Limit	Lower Weight	Upper Weight	Importance
**A:Temperature**	is in range	25	75	1	1	3
**B:Ratio**	maximize	100	150	1	1	3
**C:Time**	minimize	1	5	1	1	3
**FRAP**	none	8.19	36.2	1	1	3
**TPC**	is in range	0.812	4.47	1	1	3

Central Composite Design

Table S6 - TPC (mg GAE/g dw) and FRAP (mg AA/L) results and the CCD predicted values according to each condition for C. crispus

Run	Factor A	Factor B	Factor C	Response FRAP	Response TPC
**1**	25	100	1	15.0	0.748
**2**	50	125	3	10.0	2.86
**3**	75	150	1	7.13	2.05
**4**	50	125	3	8.31	2.96
**5**	75	125	3	10.0	1.41
**6**	25	150	1	12.0	2.45
**7**	25	125	3	12.6	2.13
**8**	50	150	3	5.51	2.99
**9**	50	125	1	12.3	1.04
**10**	25	150	5	15.1	5.68
**11**	50	100	3	7.81	2.52
**12**	75	100	1	6.89	1.34
**13**	75	100	5	8.07	2.60
**14**	75	150	5	8.09	3.77
**15**	50	125	3	10.8	2.36
**16**	25	100	5	14.2	1.81
**17**	50	125	5	6.58	2.75

- Response 1: FRAP

ANOVA for Reduced Quadratic model

Table S7 - ANOVA for Reduced Quadratic model for C. crispus

Source	Sum of Squares	df	Mean Square	F-value	p-value	
**Model**	102	2	50.9	14.6	0.0004	significant
**A-Temperature**	82.7	1	82.7	23.7	0.0002	
**A^2**	19.1	1	19.1	5.46	0.0348	
**Residual**	48.9	14	3.49			
**Lack of Fit**	45.7	12	3.81	2.39	0.3331	not significant
**Pure Error**	3.19	2	1.60			
**Cor Total**	151	16				

Fit Statistics

Table S8 - Fit Statistics of ANOVA for Reduced Quadratic model for C. crispus

**Std. Dev.**	**1.87**		**R^2**	**0.676**
**Mean**	10.0		**Adjusted R^2**	0.629
**C.V. %**	18.6		**Predicted R^2**	0.540
			**Adeq Precision**	7.33

Model Graph

The Model Graph provides a comprehensive visualization of the Central Composite Design's linear model for C. crispus. Figure S2. showcases the relationship between the FRAP values (mg AA/ L). and the significant factor AB (temperature × biomass:solvent ratio).

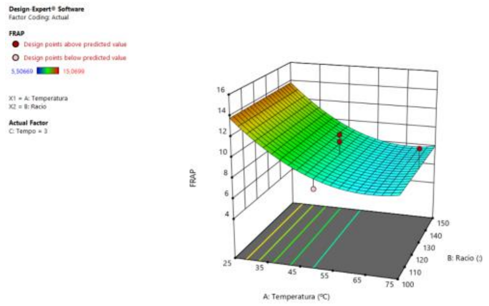

Figure S2 - Model graph for the factor AB (temperature × biomass:solvent ratio) of ANOVA for Reduced Quadratic model for C. crispus

- Response 2: TPC

ANOVA for Quadratic model

Table S9 - ANOVA for Quadratic model for C. crispus

Source	Sum of Squares	df	Mean Square	F-value	p-value	
**Model**	18.6	9	2.06	6.25	0.0123	significant
**A-Temperature**	0.266	1	0.2661	0.8052	0.3994	
**B-Ratio**	6.27	1	6.27	18.97	0.0033	
**C-Time**	8.07	1	8.07	24.42	0.0017	
**AB**	1.70	1	1.70	5.14	0.0578	
**AC**	0.2149	1	0.2149	0.6503	0.4465	
**BC**	0.8693	1	0.8693	2.63	0.1489	
**A^2**	0.2931	1	0.2931	0.8868	0.3777	
**B^2**	1.15	1	1.15	3.49	0.1040	
**C^2**	0.1136	1	0.1136	0.3436	0.5761	
**Residual**	2.31	7	0.3305			
**Lack of Fit**	2.11	5	0.4220	4.14	0.2059	not significant
**Pure Error**	0.2039	2	0.1019			
**Cor Total**	20.90	16				

Fit Statistics

Table S10 - Fit Statistics of ANOVA for Quadratic model for C. crispus

**Std. Dev.**	**0.5749**		**R^2**	**0.8893**
**Mean**	2.44		**Adjusted R^2**	0.7469
**C.V. %**	23.57		**Predicted R^2**	0.0682
			**Adeq Precision**	10.4998

Model Graph

The Model Graph provides a comprehensive visualization of the Central Composite Design's linear model for *C. crispus*. Figure S3. showcases the relationship between the TPC values (mg GAE/g dw). and the significant factor AB (temperature × biomass:solvent ratio).

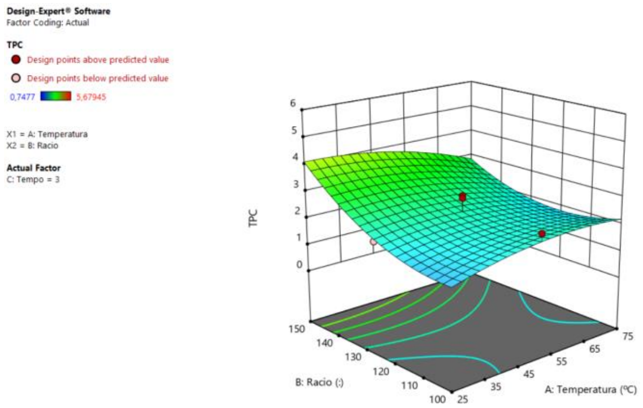

Figure S3 - Model graph for the factor AB (temperature × biomass:solvent ratio) of ANOVA for Quadratic model for C. crispus

Constrains

Table S11 presents the constraints to identify the optimal extraction conditions. These constraints were systematically applied during the CCD process to ensure that the extraction conditions remained within specified boundaries. thereby allowing us to explore the most favorable combinations of variables.

Table S11 - Constrains of the optimal conditions of ANOVA for Quadratic model for C. crispus

Name	Goal	Lower Limit	Upper Limit	Lower Weight	Upper Weight	Importance
**A:Temperature**	minimize	25	75	1	1	3
**B:Ratio**	maximize	100	150	1	1	3
**C:Time**	is in range	1	5	1	1	3
**FRAP**	is in range	7.33766	20.0806	1	1	3
**TPC**	is in range	0.7477	5.67945	1	1	3
